# Editorial: Herbal Medicines in Pain Management

**DOI:** 10.3389/fphar.2022.955743

**Published:** 2022-07-06

**Authors:** Wen-Long Hu, Yu-Chiang Hung, Ming-Yen Tsai, Hung-Rong Yen, Domenico V. Delfino

**Affiliations:** ^1^ Department of Chinese Medicine, Kaohsiung Chang Gung Memorial Hospital and Chang Gung University College of Medicine, Kaohsiung, Taiwan; ^2^ Fooyin University College of Nursing, Kaohsiung, Taiwan; ^3^ Kaohsiung Medical University College of Medicine, Kaohsiung, Taiwan; ^4^ China Medical University College of Chinese Medicine, Taichung, Taiwan; ^5^ Integration of Chinese and Western Medicine, China Medical University Hospital, Taichung, Taiwan; ^6^ Section of Pharmacology, Department of Medicine and Surgery, University of Perugia, Perugia, Italy

**Keywords:** herbal medicines, ethnopharmacology, musculoskeletal pain, neuropathic pain, cancer pain, acute pain, chronic pain

## Background

Pain as a global public health priority, is an unpleasant sensory and emotional experience associated with, or resembling that associated with, actual or potential tissue damage (Raja et al., 2020). An estimated 20% of adults worldwide suffer from pain, and 10% are newly diagnosed with chronic pain each year (Goldberg and McGee, 2011). The lack of evidence for the outcomes of most of the interventions providers do for patients and access to multidisciplinary care are preeminent crises in pain management today. Current pain management strategies rely primarily on anti-inflammatory and analgesic non-steroidal anti-inflammatory drugs (NSAIDs). These medicines are the first line of treatment for mild or moderate pain, then, if they fail, through the more efficient opioids. However, opioids are troubled by adverse effects and drug addiction. Opioids can cause sedation, nausea/vomiting, constipation, tolerance, physical dependence, and respiratory depression (Crofford, 2010). Therefore, finding alternatives, from botanicals in particular, for pain management is essential.

Far from the edges of modern health care, many patients with chronic pain often use many complementary therapies. More and more, these unconventional treatments have undergone the same rigorous investigation as all contemporary evidence-based medicine practices (Simpson, 2015). A survey conducted in 16 countries revealed that 67% of participants with chronic pain sought other forms of treatment besides medication (Breivik et al., 2006). Herbal medicines are the most commonly used for the treatment of pain in traditional Chinese medicine (TCM) and other traditional medicine systems worldwide. This Research Topic aims to explore herbs and herbal formulations for the treatment of pain, including acute, chronic, or cancer pain, and their mechanisms.

## Overview of the Articles Included in This Research Topic

Pain is a frequent symptom in spinal cord injury (SCI) and this results in a low quality of life for these patients. The problem is substantial since, in the world, from 250,000 to 500,000 patients in a year suffer from SCI (40–80 cases per million), with a total cost per patient 1.1–4.6 million dollars. Conventional treatment consists of corticosteroids, immobilization of the spine, surgery, anticoagulant prophylaxis, and pain management. The most widely used pain medications are opioids, gabapentinoids, α-adrenergic antagonists, antidepressants, and anticonvulsants with not always satisfactory results. Since cannabinoids are used to produce analgesic effects through the activation of TRPA1, TRPV1, TRPV2, TRPV4 receptors and other G-protein coupled receptors, Tsai et al., addressed the role of these plant products in reducing the pain of patients with SCI. Through a systematic review of data searched in the PubMed, EMBASE, Scopus, Cochrane, Web of Science, and ClinicalTrials.gov for relevant randomized controlled trials (RCTs), following the instructions of the Preferred Reporting Items for Systematic reviews and Meta-Analyses (PRISMA). The Cochrane ROB 2.0 and Grading of Recommendations, Assessment, Development, and Evaluations (GRADE) systems were used to assess the quality of studies and evidence. 417 patients with SCI (from a total of 9,500 documents) were included in the analysis. Compared to these numbers, there were no significant differences between patients treated with cannabinoids and those treated with placebo in pain reduction. While the analysis revealed a high probability of adverse effects. Tsai et al., suggest that the use of cannabinoids may not be beneficial in the treatment of patients suffering from SCI while there may be an increase in adverse events such as dizziness, drowsiness and dysgeusia. However, further studies, perhaps more targeted, are needed to completely rule out the goodness of cannabinoid use in these patients.

Chronic pain is the most prevalent health problems in the world. Jiang et al. introduced some analgesic alkaloids derived from TCM, including tetrahydropalmatine, aloperine, oxysophocarpine, matrine, sinomenine, ligustrazine, evodiamine, brucine, tetrandrine, Stopholidine, and lappaconitine, focusing on their mechanisms and potential clinical applications. Jiang et al. applied the concept of drug-cloud (dCloud) theory for better depicting the mechanism of these alkaloids. dCloud showed the complete therapeutic range of multi-target analgesics with two dimensions, which are “direct efficacy,” including inhibition of ion channels, activating gamma-aminobutyric acid/opioid receptors, direct inhibition of pain signal; and “background efficacy,” including diminishing neuronal inflammation/oxidative stress, inhibiting glial cell activation, recovering the balance between excitatory and inhibitory neurotransmission, healing the underlying causes of chronic pain. Empirical evidence suggests that drug combinations are favorable to 30%–50% of patients with chronic pain. Subsequently, Jiang et al. also explained the therapeutic effect of analgesic compositions containing Chinese medicine-derived analgesic alkaloids, which may help to model alternative drug discovery. As natural compounds, TCM analgesic alkaloids generally have moderate potency, but they have low long-term side effects, particularly in comparison with opioids (Du et al., 2016), thus are potential drug candidates.

Chronic administration of morphine often leads to the development of tolerance, which leads to increased doses, thereby reducing the viability of long-term clinical opioid use. Extensive research work has been done to explain the role of various neurotransmitters, channels, and receptor systems in the development of morphine tolerance. Recent reports have shown that opioids alter the properties of neurons expressing MORs and nociceptive circuits at the level of the dorsal root ganglia (DRG) and spinal cord dorsal horn (Christie, 2008; Rivat and Ballantyne, 2016). Yokukansan (YKS) is a traditional Kampo medicine to treat emotional irritability, neurosis, and insomnia. Ohashi et al. study has used the hot plate test after YKS morphine to assess the rats’ tolerance of analgesic effect of morphine injection. The results found that the YKS administration may inhibit the development of morphine tolerance *via* suppressing the hyper-function of the presynaptic transmission of DRG neurons. YKS also can inhibit the activation glial cells in the spinal cord during long-term morphine treatment, to block the release of cytokines (i.e., IL-1β, IL-6, TNF-α) and spinal cord inflammatory immune responses. Neither Morphine nor YKS changed the expression of postsynaptic proteins, such as NMDA glutamate receptors (NR2A, 2B), AMPA glutamate receptors (GluR 2/3/4) and PSD95, nor did they change the expression of inhibitory GABAergic presynaptic marker protein (GAD 65/67). The findings strongly support that presynaptic protein level has an intrinsic role in the development of morphine antinociceptive tolerance.

In South Africa, traditional medicine remains top priority for pain relief and inflammatory related diseases. Aremu and Pendota focuses on analyzing trends and patterns in plants used in South African folk medicine to alleviate pain and inflammatory-related diseases. A large-scale search was implemented using different scientific databases and popular ethnobotanical literature focusing on South African ethnobotany. According to the systematic analysis, 38 sources were chosen to generate a list of 495 plants from 99 families believed to be medicines for pain and inflammatory-related diseases (e.g., headache, toothache, backache, menstrual pain, and rheumatism) between different races in South Africa. Fifty five percent of the 38 studies were recorded in three provinces, i.e., KwaZulu-Natal, Limpopo, and Western Cape. The most popular plants applied for pain and inflammatory-related diseases in South Africa were *Ricinus communis* L., *Aloe ferox* Mill., *Pentanisia prunelloides* subsp. latifolia (Hochst.) Verdc., *Dodonaea viscosa* Jacq var. angustifolia (L.f) Benth., (L.) W.T.Aiton.*Ruta graveolens* L., and *Solanum aculeastrum* Dunal. The top five plant families represented were Asteraceae (13%), Fabaceae (8%), Apocynaceae (4.3%), Asparagaceae (4%), and Lamiaceae (4%). An approximate 54% of recorded plants are woody (trees and shrubs) in nature, with leaves (27%) and roots (25%) the most important plant parts. The use of plants to relieve pain and inflammatory-related diseases is still popular in South African folk medicine. Aremu and Pendota bring some useful information such as plant parts used, preparation methods, and recipes for most of the identified plants in South Africa.

Endometriosis is an estrogen-dependent gynecological inflammatory disorder that can cuase infertility and recurrent pelvic pain. Gao et al. performed a systemic review and meta-analysis to investigate the efficacy and safety of Salvia miltiorrhiza-containing Chinese herbal medicine (CHM) in combination with gonadotropin-releasing hormone agonist (GnRH-a) to treat postoperative endometriosis. In comparison with the control group, the combination of CHM containing Salvia miltiorrhiza and GnRH-a group had significant advantage in reducing the relapse of endometriosis and increasing the pregnancy rate. Likewise, there was a positive effect of CHM containing Salvia miltiorrhiza in combination with GnRH-a on CA-125 serum levels. In addition, side effects were significantly reduced in this group. This meta-analysis suggests that CHM containing Salvia miltiorrhiza can be used as a complementary therapy for postoperative endometriosis management. CHM containing Salvia miltiorrhiza seems to be a potential drug to improve clinical efficacy and reduce GnRH-a side effects. However, because of low quality of most of the enrolled studies, further large-scale, high-quality, rigorous RCTs are needed to strengthen the results.

Inflammatory cartilage degeneration of joints, mainly knee, hips and hands, is osteoarthritis (OA). Existing OA treatment strategies aim at pain relief, molecular targeting, cartilage regeneration and surgery. Herbal mineral formula Peedanil Gold (PN-G) has been used to treat arthralgia and inflammation. Balkrishna et al. investigated the anti-osteoarthritis effectiveness of PN-G in an OA rat model. PN-G ameliorated clinical and Kellgren & Lawrence scores; and salvaged OA rats from hyperalgesia and allodynia. Furthermore, PN-G improved joint inflammation and eliminated *in vivo* osteoarthritis by efficacious cartilage regeneration regarding pathological, radiological and histopathological measures. PN-G also decreased the levels of IL-6 and IL-1 beta, in a dose-dependent manner, in inflamed human macrophage THP-1 cells, therefore, reiterating its anti-inflammatory property at cell-safe concentrations. Ultra High performance liquid chromatography (UHPLC) revealed the presence of several analgesic and anti-inflammatory plant compounds in PN-G, such as ellagic acid, guggulsterone E, guggulsterone Z, 5-(hydroxymethyl) furfural, corilagin, cinnamic acid, ferulic acid, gallic acid and protocatechuic acid. Balkrishna et al. concisely illustrated that PN-G is able to relieve the clinical symptoms of OA as measured by cartilage oligomeric matrix protein, an established OA serum biomarker.

Cancer pain is major issue in cancer therapy that impacts on patient quality of life and survival-related outcomes. East Asian herbal medicine (EAHM) monotherapy reduces pain management related side effects in cancer patients. In addition, EAHM in combination with conventional treatment can help patients with cancer pain improve response rate, reduce pain severity, improve pain-related functional status, and modulate opioid use. However, it is difficult to draw conclusions about the effectiveness and safety of EAHM monotherapy because of lacking methodological quality and quantity of studies. According to the core herbal combination patterns derived from the review by Jo et al., four herbal combination pairs may be helpful for cancer pain, as they are frequently used apparently for cancer patients in East Asia. Therefore, they are deemed worthy of follow-up studies to clarify their role and effects.
